# Luteolin Alleviates Inflammation Induced by *Staphylococcus aureus* in Bovine Mammary Epithelial Cells by Attenuating NF-κB and MAPK Activation

**DOI:** 10.3390/vetsci12020096

**Published:** 2025-01-27

**Authors:** Yingfang Guo, Jinxin Zhang, Ting Yuan, Cheng Yang, Qingqing Zhou, Aftab Shaukat, Ganzhen Deng, Xiaoyan Wang

**Affiliations:** 1 School of Physical Education, Wuhan Business University, Wuhan 430056, China; 20231109@wbu.edu.cn; 2 Department of Clinical Veterinary Medicine, College of Veterinary Medicine, Huazhong Agricultural University, Wuhan 430056, China; zhangjinxin@webmail.hzau.edu.cn (J.Z.); cheng.yang@webmail.hzau.edu.cn (C.Y.); dgz@mail.hzau.edu.cn (G.D.); 3 College of Animal Science and Technology, Chongqing Three Gorges Vocational College, Chongqing 404155, China; ytcqsx@163.com; 4 College of Veterinary Medicine, Yunnan Agricultural University, Kunming 650201, China; zhouqingqing@ynau.edu.cn; 5 Department of College of Veterinary Medicine, South China Agricultural University, Guangzhou 510642, China; dr.aftabshaukat@scau.edu.cn

**Keywords:** luteolin, *Staphylococcus aureus*, TLR2, NF-κB pathway, MAPK pathway, mammary epithelial cells

## Abstract

The internalization of *S. aureus* in bMECs is a major pathogenic mechanism leading to mastitis, causing significant economic losses in the dairy industry. Numerous plants contain Lut, a natural flavonoid with anti-inflammatory and antioxidant properties. However, little is known about Lut’s ability to reduce inflammation caused by *S. aureus* in bMECs. This research aimed to evaluate the mechanism by which Lut reduces *S. aureus*-induced inflammation in bMECs. Through GO and KEGG enrichment analysis, researchers analyzed the differentially expressed genes in bMECs infected with *S. aureus* in NCBI GEO (GSE139612) and also analyzed the targets of Lut predicted by various online platforms. These studies identified two overlapping signaling pathways, the NF-κB and the MAPK pathways. We stimulated bMECs with *S. aureus* for two hours and then added Lut for ten hours, with a total duration of twelve hours. The expression levels of TLR2-MyD88-TRAF6 components, inflammatory cytokines, and protein phosphorylation associated with the MAPK and NF-κB signaling pathways were then assessed. Based on all of the results, Lut inhibited the generation of inflammatory cytokines in bMECs that were induced by *S. aureus* through the TLR2, NF-κB, and MAPK signaling pathways. This process might account for the anti-inflammatory properties of Lut.

## Simple Summary

Infection of udder tissue by pathogens is the main cause of bovine mastitis. Mastitis results in substantial financial losses for the dairy industry by reducing milk production, compromising the quality of dairy products, and elevating mortality rates. The most common pathogen causing mastitis is Staphylococcus aureus. The initial line of defence of mammary tissue consists of immune cells and mammary epithelial cells. Antibiotics are typically used to treat mastitis nowadays. However, the overuse of these medications has increased bacterial resistance, endangering human health. As a result, creating extremely efficient treatments with minimal toxicity and residue is essential. Research indicates that luteolin, a plant extract, has a number of advantageous qualities, including neuroprotective, anti-inflammatory, antioxidant, and antitumor actions. We examined the potential of luteolin as a therapeutic agent for the treatment of bovine mammary epithelial cells in the context of Staphylococcus aureus infection, thereby establishing a foundation for future management strategies for bovine mastitis. It is anticipated that this extract will find widespread application in the future.

## 1. Introduction

Mastitis in dairy cows is widely recognized as the most prevalent and economically impactful disease affecting global dairy herds, resulting in substantial financial losses [1]. An estimated 200 distinct species of microorganisms have been identified as potential causative agents of mastitis in cows. Among these, bacteria, fungi, and viruses are the predominant pathogenic microbes associated with this condition [2]. Among them, Staphylococcus aureus (*S. aureus*) is one of the most common causes of bovine mastitis worldwide [3]. Antibiotics are now used to treat bovine mastitis induced by *S. aureus*, but their residues pose serious risks to human health [4]. Therefore, veterinarians and related professionals have invested a lot of energy in strengthening the local innate immune system to defend against dangerous microorganisms.

In order to identify harmful pathogens and regulate inflammation, mammary epithelial cells express pathogen recognition receptors (PRRs), which promote the synthesis of cytokines [5,6]. Innate immune responses are significantly influenced by toll-like receptors (TLRs) [7]. Myeloid differentiation factor 88 (MyD88), which consists of two primary do-mains—the C-terminal Toll/IL-1R (TIR) domain and the N-terminal death domain—must be activated intracellularly for TLR2 signaling to be successful. The TIR domain of MyD88 interacts with the TIR domain of the TLR receptor during ligand attachment. The death domain of IL-1 receptor-associated kinase (IRAK), a serine/threonine kinase, is located in its N-terminal region and directly interacts with the death domain of MyD88. IRAK becomes phosphorylated, which attracts TNF receptor-associated factor 6 (TRAF6). When TLR2 interacts with *S. aureus*, it activates intracellular signaling pathways, such as nuclear factor kappa-B (NF-κB) and mitogen-activated protein kinase (MAPK), leading to inflammatory reactions [8,9,10]. In rat mammary tissue, TLR2 activation after *S. aureus* stimulation stimulates the phosphorylation of the MAPK (p38, ERK, JNK) and NF-κB (IκBα, p65) signaling pathways [11]. A number of important inflammatory mediators, including cytokines and chemokines, are synthesized through NF-κB and MAPK signaling [12]. Potential treatments for *S. aureus*-induced mastitis may be evaluated using a wealth of information from natural compounds with a wide range of biological traits, including antioxidant, anti-inflammatory, and anticancer activities.

Luteolin (Lut) is a member of the family of natural flavonoid compounds [13]. There are numerous varieties of flowers, herbs, vegetables, and spices from which it can be derived. Lut was found to inhibit the excessive release of proinflammatory markers in previous studies [14,15]. It has been demonstrated that Lut significantly reduces the phosphorylation of the NF-κB pathway in endometritis caused by *S. aureus* [16]. Lut may simultaneously reduce IL-1β-induced THP-1 adhesion to ARPE-19 cells and inhibit inflammatory responses by blocking the activation of the MAPK and NF-κB pathways [17]. A particular dose of Lut has been shown to cause DNA damage and apoptosis [18], and it may prevent the NF-κB pathway from producing inflammatory mediators in RAW264.7 cells infected with the pseudorabies virus [19]. The effect of Lut on bovine mastitis is still unknown, though. This work aims to improve our knowledge of the signaling pathways linked to Lut and investigate its function in the inflammatory response that *S. aureus* induces in bovine mammary epithelial cells (bMECs).

## 2. Materials and Methods

### 2.1. Chemicals and Reagents

Lut was obtained from Shanghai Yuanye Bio-Technology Co., Ltd., located in Shanghai, China. *S. aureus* (ATCC 25923), which can cause mastitis in dairy cows, was acquired via Shanghai Fuxiang Biotechnology Co., Ltd., located in Shanghai, China [20]. The Nanjing Jiancheng Bioengineering Institute in Nanjing, China is the source of the cytokine ELISA kits (IL-1β, IL-6, and TNF-α). RT-qPCR and CCK8 reagents were purchased from Yeasen Biotechnology, Shanghai, China. Primary and secondary antibodies (TLR2, phospho-IκBα (p-IκBα), phospho-p65 (p-p65), phospho-JNK (p-JNK), phospho-ERK (p-ERK), phospho-p38 (p-p38), and β-actin) were procured from Cell Signaling Technologies, Beverly, MA, USA.

### 2.2. Bioinformatics Analysis

Data related to *S. aureus*-induced inflammatory responses of bMECs were downloaded from GEO databases for the current investigation (http://www.ncbi.nlm.nih.gov/geo/ accessed on 18 November 2024, GSE139612). After identifying differentially expressed genes (DEGs), we performed enrichment analyses utilizing the Kyoto Encyclopedia of Genes and Genomes (KEGG) database (https://www.kegg.jp/) and the Gene Ontology (GO) resource (http://geneontology.org/). The Traditional Chinese Medicine Systems Pharmacology database (TCMSP, https://old.tcmsp-e.com/tcmsp.php accessed on 18 November 2024) was used to assess the druggability of Lut. The SwissTarget Prediction (http://www.swisstargetprediction.ch/) and Herb (http://herb.ac.cn) were used to identify possible targets of Lut. Furthermore, GO and KEGG studies were used to perform enrichment analysis of targets of Lut.

### 2.3. High-Pressure Liquid Chromatography (HPLC) Analysis

Lut was dissolved in methanol to form solution A. Then, solution A was combined with a 0.05% trifluoroacetic acid aqueous solution to prepare mobile phase A. Mobile phase B consisted of acetonitrile, as previously described in the HPLC procedure [21]. The Lut solution was added into a Sinochrom ODS-BP chromatographic column (4.6 × 250 mm, 5 μm) at a detection wavelength of 346 nm.

### 2.4. Cell Counting Kit-8 (CCK-8)

The effect of Lut on MAC-T cell viability was investigated using CCK-8. First, 100 μL of media was used to culture the cells in an incubator at 37 °C with 5% CO_2_. A density of 1 × 10^5^ cells per well was used for seeding in a 96-well plate. When the cells reached 90% growth, Lut (2.5 μg/mL, 5 μg/mL, 7.5 μg/mL, and 10 μg/mL) was added, and they were incubated for 12 h. After adding 10 μL of CCK-8 reagent to each well, the cells were incubated for 4 h at 37 °C in the dark. A microplate reader was used to measure the optical density (OD) at 450 nm.

### 2.5. Cell Culturing and Treatment Groups

The cells were grown using the bovine mammary epithelial cell line MAC-T, which was acquired from the American Type Culture Collection (ATCC, Manassas, VA, USA), in compliance with previous studies [22]. To sustain the cell culture, a 1:1 ratio of DMEM/F-12 mix supplied by Gibco (Waltham, MA, USA) was mixed with 10% fetal bovine serum. The cultivated cells were kept at 37 °C in a humidified environment with 5% CO_2_.

Lysogenic broth (LB) was used to cultivate *S. aureus*, and the LB plate counting method was used to determine the colony-forming units (CFUs) [23]. MAC-T cells in a suitable suspension state were aseptically seeded into each well of a 6-well plate. The multiplicity of infection (MOI) for *S. aureus* was set at 10. The introduction of *S. aureus* into five of the wells occurred after the cell density reached 60% to 70%. Following a two-hour incubation period, three groups were administered varying amounts of Lut. Dexamethasone (DEX), as a therapeutic drug for comparison, was added to one group. The six groups were as follows:

(1) Control group (C.G.): without any treament;
(2) *S. aureus* group (S.A.G.): only added *S. aureus* for 12 h;
(3) *S. aureus* + Lut group (2.5 μg/mL): added *S. aureus* for 2 h and then added Lut (2.5 μg/mL) for a total of 12 h;
(4) *S. aureus* + Lut group (5 μg/mL): added *S. aureus* for 2 h and then added Lut (5 μg/mL) for a total of 12 h;
(5) *S. aureus* + Lut group (7.5 μg/mL): added *S. aureus* for 2 h and then added Lut (7.5 μg/mL) for a total of 12 h;
(6) *S. aureus* + DEX group (DEX) (5 μg/mL): added *S. aureus* for 2 h and then added DEX (5 μg/mL) for a total of 12 h.

### 2.6. Enzyme-Linked Immunosorbent Assay (ELISA) Assay

The quantities of cytokines generated by *S. aureus*-stimulated MAC-T cells at varying Lut concentrations were quantitatively detected through ELISA. The control group and the dexamethasone intervention group stimulated by *S.* aureus were also tested. As directed by the manufacturer’s instructions, the amounts of TNF-α, IL-1β, and IL-6 were quantitatively measured using an ELISA kit.

### 2.7. Real-Time Quantitative Polymerase Chain Reaction (RT-qPCR)

Total RNA was extracted from MAC-T cells using the TRIzol reagent (Solarbio, Bei-jing, China). An ultra-differential spectrophotometer Q5000 was used to assess the purity of the RNA, following the manufacturer’s instructions. After detecting RNA concentration, qPCR reagent (gDNA digester+), Hieff ^®^ qPCR SYBR ^®^ Super mixing, and Hieff ^®^ III First strand cDNA synthesis were mixed. To determine the expression levels of the target genes, RT-qPCR was employed. Table 1 lists the primer sequences that were employed in this investigation. For quantitative analysis, the 2^−ΔΔCt^ technique [24] was used, with the GAPDH gene as the internal reference gene.

### 2.8. Western Blotting

The protein content of the total protein extracted from MAC-T cells using RIPA rea-gents was measured using the BCA protein detection kit in accordance with the manufacturer’s instructions. Then, 10% sodium dodecyl sulfate polyacrylamide gel electrophoresis (SDS-PAGE) was used to separate the protein. The gel was then applied to the PVDF membrane to transfer the mold. The membrane was then incubated for two hours in 5% skim milk powder. The membrane was then subjected to three TBST washes. The membrane was then exposed to the main antibody for the whole night at 4 °C. After that, the membrane was cleaned once again using the proper amount of TBST. After the second antibody was added, the membrane was incubated for an hour at 37 °C.

### 2.9. Data Analysis

GraphPad Prism 8 software (San Diego, CA, USA) was used to conduct the statistical analysis. Data were expressed as mean ± SEM from three separate experiments. After per-forming the normality test, Student’s *t*-tests or one-way ANOVA were used to analyze group differences. Significance levels were indicated as # *p* < 0.01, * *p* < 0.05, and ** *p* < 0.01 when compared to the control group, the *S. aureus* group, and the *S. aureus* group, respectively.

## 3. Results

### 3.1. DEG Study of Inflammation Caused by S. aureus from the GEO Database

**Figure 1:**
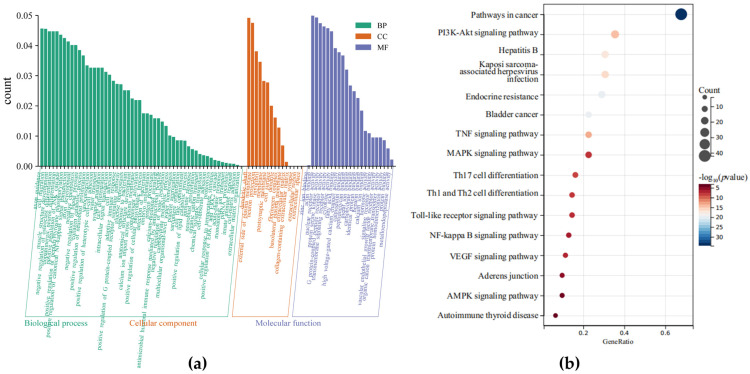
DEG analysis of the GEO database (GSE139612) for *S. aureus*-induced mastitis. (**a**) GO enrichment analysis; (**b**) KEGG enrichment analyses of DEGs.

Significant changes in gene expression occur when host infection events take place. In this investigation, we used data from the GEO database (GSE139612) [25] to investigate the alterations in gene expression brought on by *S. aureus* infection with bMECs. DEGs in the dataset were identified using GEO2R [26], and GO and KEGG analyses were used for enrichment analysis (Figure 1). Numerous pathways, including NF-κB and MAPK, were linked to these DEGs.

### 3.2. Network Pharmacology of Lut

**Figure 2:**
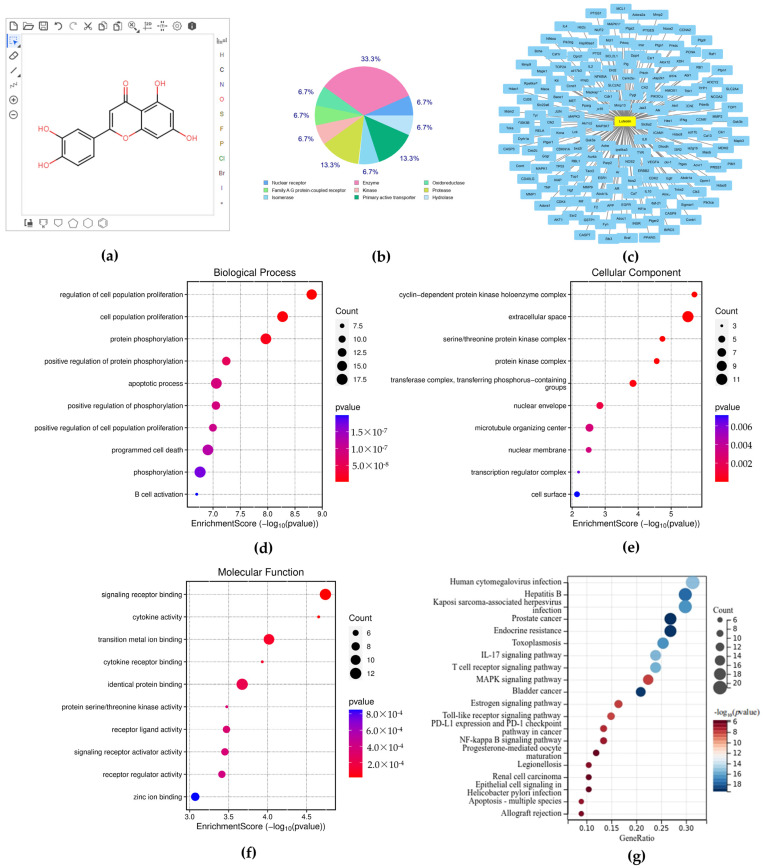
Analysis of Lut using network pharmacology. (**a**) The chemical structure of the Lut molecule; (**b**) the target classes of Lut; (**c**) the potential targets of Lut were identified using SwissTarget Prediction; (**d**–**f**) GO analysis of targets, encompassing molecular activities, biological processes, and cellular components; (**g**) KEGG analysis of targets.

Lut possesses 179 potential targets and high medicinal properties, as seen in Figure 2. The targets were discovered to be enriched in inflammatory, immunological, and other characteristics after GO and KEGG enrichment analysis. NF-κB and MAPK were the signaling pathways in which both DEGs in GSE139612 and Lut targets were involved, according to an enrichment analysis. Thus, we would continue to investigate the MAPK and NF-κB signaling pathways to elucidate their roles in the biological processes related to Lut’s functions.

### 3.3. HPLC and CCK 8 Analysis

**Figure 3:**
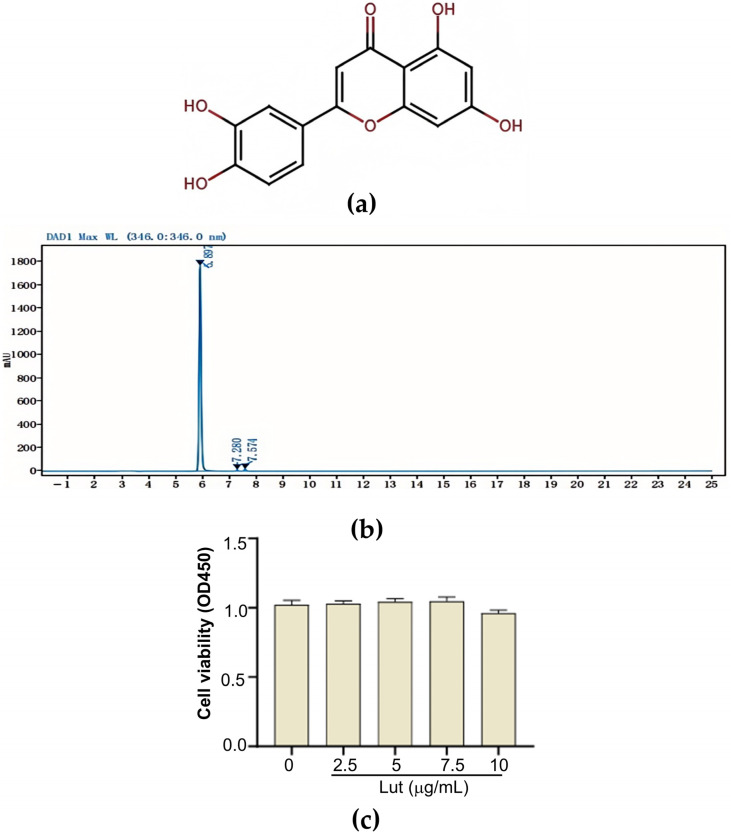
MAC-T cell activity was unaffected by purified Lut. (**a**) The chemical structure of Lut; (**b**) purity of Lut determined through HPLC; (**c**) the effect of Lut on MAC-T cell viability was assessed through CCK-8.

The purpose of the HPLC analysis was to guarantee the quality and purity of Lut, which was essential for the dependability of ensuing studies examining its impact on MAC-T cells. The extract was found to be highly pure (Figure 3b). CCK-8 was used to assess the cytotoxicity of Lut. The CCK-8 assay results (Figure 3c) showed that Lut did not negatively impact MAC-T cells at concentrations lower than 7.5 μg/mL.

### 3.4. Lut Reduced Pro-Inflammatory Cytokine Secretion from MAC-T Cells During S. aureus Infection

**Figure 4:**
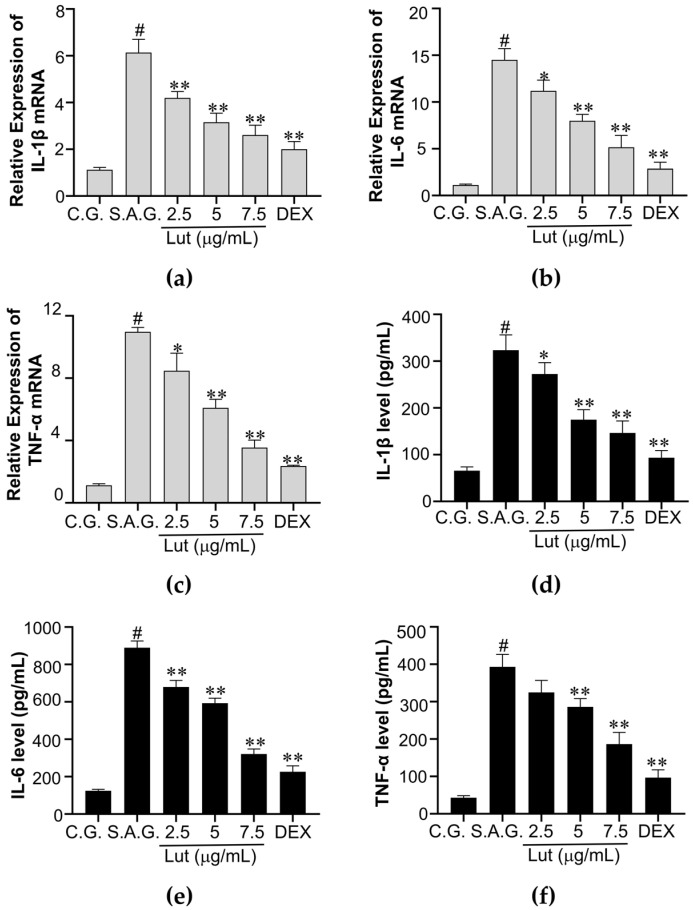
The MAC-T cells infected with *S. aureus* were prevented from releasing cytokines by Lut. (**a**–**c**) Using GAPDH as the internal reference gene, RT-qPCR was utilized to assess the mRNA of IL-1β, IL-6, and TNF-α; (**d**–**f**) levels of IL-1β, IL-6, and TNF-α were measured through ELISA; the control group, the *S. aureus* stimulation group, and the combination group of *S. aureus* and dexamethasone were designated as C.G., S.A.G., and DEX, respectively. The findings were expressed using the means ± SEM of three separate studies. The markers of statistical significance were as follows: # *p* < 0.01, * *p* < 0.05, and ** *p* < 0.01 when compared to the control group, the *S. aureus* group, and the *S. aureus* group, respectively.

Cytokine expression levels were measured using RT-qPCR and ELISA. The results showed that pro-inflammatory cytokine mRNA expression levels were significantly higher in MAC-T cells infected with *S. aureus* and significantly lower when Lut was added as an intervention. Compared with other Lut dosages, the lowest mRNA expression levels were obtained at a Lut dosage of 7.5 μg/mL compared with other Lut dosages (Figure 4a–c). For TNF-α, IL-1β, and IL-6 levels, the RT-qPCR trend matched the ELISA results (Figure 4d–f). Lut (7.5 μg/mL) and DEX had comparable impacts on the levels of cytokine expression.

### 3.5. Lut Reduced TLR2 Expression in S. aureus-Infected MAC-T Cells

**Figure 5:**
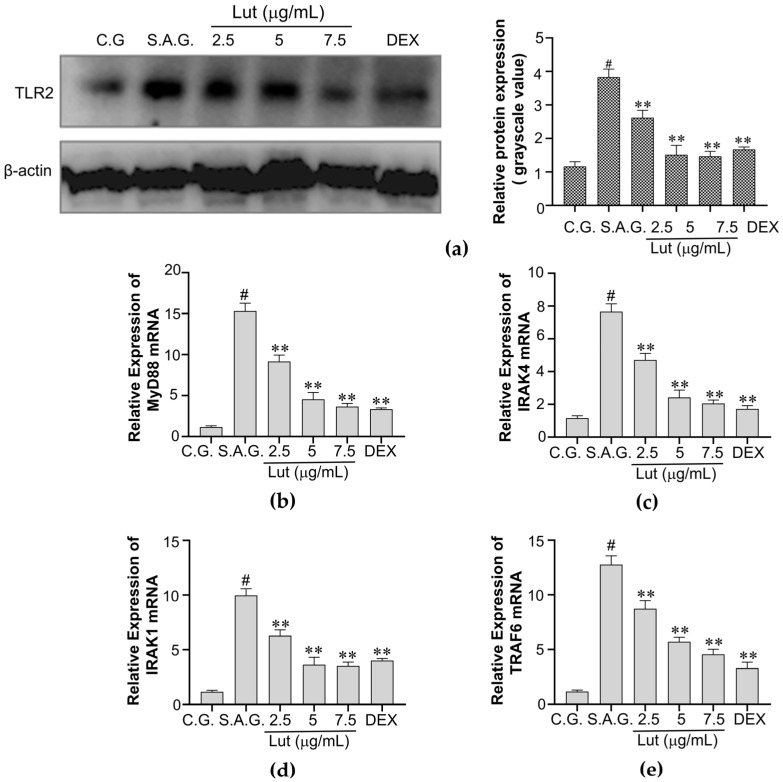
Western blotting was used to measure the levels of protein expression. (**a**) β-actin served as a control, and the grayscale value of TLR2 protein expression was computed using ImageJ (1.46r) software. (**b**–**e**) RT-qPCR was utilized to ascertain the mRNA expression of MyD88, IRAK4, IRAK1, and TRAF6 utilizing GAPDH as an internal reference gene. The control group, the *S. aureus* stimulation group, and the combination group of *S. aureus* and dexamethasone were designated as C.G., S.A.G., and DEX, respectively. The findings were expressed using the means ± SEM of three separate studies. The markers of statistical significance were as follows: # *p* < 0.01, and ** *p* < 0.01 when compared to the control group, the *S. aureus* group, and the *S. aureus* group, respectively.

TLR2 initiates the chain reaction of inflammatory signals after detecting *S. aureus* [27]. We found that TLR2 expression was greater in the *S. aureus* group than in the control group. When Lut was added, it was found that TLR2 expression was lower than that in S.A.G. The relative expression levels of TLR2 in various groups are displayed in Figure 5a. The mRNA expression of IRAK4, IRAK1, and TRAF6 significantly increased in *S. aureus*-induced MAC-T cells; however, Lut abolished these effects. Variations of mRNA expression of MyD88, IRAK4, IRAK1, and TRAF6 under various circumstances are displayed in Figure 5b. In conclusion, Lut has been shown to down-regulate the TLR2 signaling pathway in MAC-T cells for the first time.

### 3.6. Lut Inhibited the Activation of NF-κB Pathway in S. aureus-Infected MAC-T Cells

**Figure 6:**
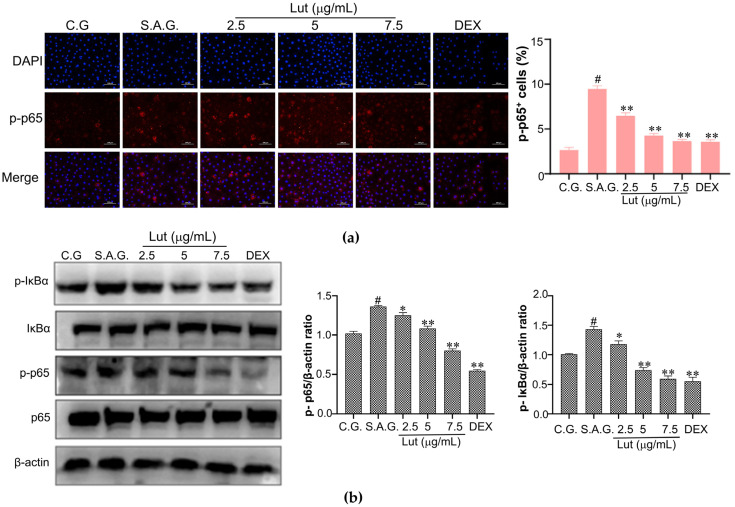
Lut inhibits proteins in the NF-κB signaling pathway from becoming phosphorylated. (**a**) The transit of the NF-κB p65 subunit from the cytoplasm to the nucleus was assessed using immuno-fluorescence microscopy. Blue fluorescence suggests nuclear staining, while red fluorescence indicates p-p65 staining. The proportion of cells that were p-p65 positive was computed using ImageJ software. (**b**) Protein expression levels of proteins implicated in the NF-κB pathway were measured through Western blotting analysis and densitometric measurements using ImageJ software, with β-actin serving as the loading control. The control group, the *S. aureus* stimulation group, and the combination group of *S. aureus* and dexamethasone were designated as C.G., S.A.G., and DEX, respectively. The findings were expressed using the means ± SEM of three separate studies. The markers of statistical significance were as follows: # *p* < 0.01, * *p* < 0.05, and ** *p* < 0.01 when compared to the control group, the *S. aureus* group, and the *S. aureus* group, respectively.

Compared to the control group, the *S. aureus* stimulation group exhibited significantly elevated phosphorylation levels of IκBα and p65. Following the addition of Lut, immunofluorescence results indicated that Lut effectively reduced the nuclear translocation of phosphorylated p65 (p-p65) induced by *S. aureus* (Figure 6a). Concurrently, the phosphorylation levels of both IκBα and p65 were markedly diminished, and that of the reduction was more pronounced at higher Lut dosages (Figure 6b).

### 3.7. Lut Inhibited the Activation of MAPK Pathway in S. aureus-Infected MAC-T Cells

**Figure 7:**
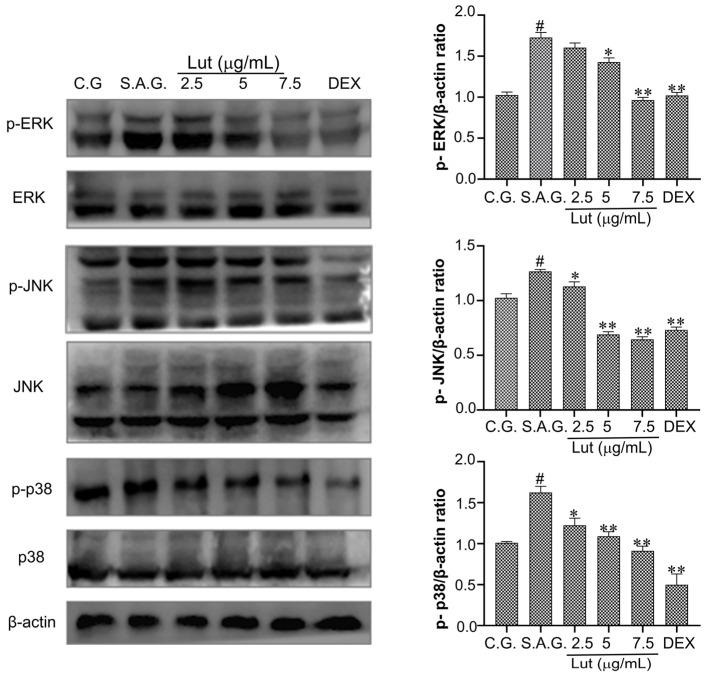
Lut prevented the phosphorylation of the MAPK pathway. Western blotting was used to measure the level of protein phosphorylation in the MAPK signaling pathway, and the protein bands’ grayscale values were examined to determine the levels of protein expression. The control group, the *S. aureus* stimulation group, and the combination group of *S. aureus* and dexamethasone were designated as C.G., S.A.G., and DEX, respectively. The findings were expressed using the means ± SEM of three separate studies. The markers of statistical significance were as follows: # *p* < 0.01, * *p* < 0.05, and ** *p* < 0.01 when compared to the control group, the *S. aureus* group, and the *S. aureus* group, respectively.

Compared to the control group, MAC-T cells infected with *S. aureus* exhibited noticeably greater phosphorylation levels of ERK, JNK, and p38 (Figure 7). Furthermore, ERK, JNK, and p38 phosphorylation levels significantly decreased as the dose of Lut rose.

## 4. Discussion

Bovine mastitis significantly impacts milk production and composition, representing one of the most costly diseases in the agricultural sector [28]. *S. aureus* is the predominant pathogen responsible for bovine mastitis, and it is capable of inducing both clinical and subclinical forms of the disease [29]. Through a detailed analysis of the GEO dataset (GSE139612), we identified that *S. aureus* infection gives rise to multiple DEGs that are enriched in inflammation-related pathways. Lut, a natural flavonoid compound derived from flowers, vegetables, and other herbs, possesses antioxidant and anti-inflammatory properties [30,31]. The cytotoxicity of Lut was evaluated in MAC-T cells, revealing that cell viability remained unaffected at concentrations below 7.5 μg/mL. Following target enrichment analysis of Lut, it was discovered that these targets were strongly enriched in the signaling pathways of MAPK, TNF-α, NF-κB, IL-17, TLRs, and so on. The pathways of enrichment of Lut targets were consistent with those in other investigations [32,33]. We discovered that the targets of Lut were involved in the same NF-κB and MAPK signaling pathways, which were the same as those of DEGs-enriched signaling pathways in GSE139612. Therefore, the purpose of this investigation is to determine whether Lut can use TLR2-NF-κB/MAPK to suppress the expression of inflammatory cytokines in MAC-T cells induced by *S. aureus*.

One of the most crucial TLRs in the immunological response triggered by *S. aureus* is TLR2 [34]. According to earlier research conducted in our lab, TLR2 effectively mediates *S. aureus* infections [35]. In the meantime, IRAK in the TLR2 signaling pathway is activated by the adaptor protein MyD88 [36]. Based on the results of previous bioinformatics work, we wanted to examine TLR2 activation and protein phosphorylation in NF-κB and MAPK signaling pathways in order to gain a deeper understanding of Lut’s role in *S. aureus*-induced MAC-T cells. TLR2 expression was measured using Western Blotting. After IRAK activation, the other adaptor protein, TRAF6, becomes phosphorylated and is attracted to IRAK in the *S. aureus* group [37,38]. Pathological conditions cause altered expression levels of pro-inflammatory cytokines, such as TNF-α, which is produced early in the inflammatory response, IL-1β, which is crucial for both adaptive and innate immunity [39], and IL-6, which is thought to be one of the main biomarkers of bovine mastitis [40]. While the appropriate proinflammatory cytokines are essential for boosting host immunity, overproduction of these cytokines can harm cells and tissue; therefore, their release in response to inflammatory stimuli must be tightly monitored [41]. Our findings showed that when *S. aureus* stimulated MAC-T cells, TLR2 expression and the pro-inflammatory cytokines IL-1β, IL-6, and TNF-α were significantly increased. These findings suggested that *S. aureus* activated MAC-T cells to produce severe inflammatory responses. Lut may reduce the inflammation of MAC-T cells by blocking the release of TNF-α, IL-1β, and IL-6, as evidenced by the sharp decline in pro-inflammatory cytokine and TLR2 after the addition of Lut.

By identifying pathogens and interacting with cells, TLR2 generates inflammatory cytokines that activate immune responses by activating downstream signaling pathways, such as NF-κB and MAPK [42]. Downstream of TLRs, IκBα and p65 activate important inflammatory signaling pathways and control the production and expression of inflammatory mediators [43]. Inflammatory events induce the IκBα protein to become quickly phosphorylated, which promotes the entry of p65 into the nucleus. Normally, IκBα and p65 are connected. The NF-κB signaling system is activated and numerous genes, including those that generate pro-inflammatory cytokines, are regulated. Consequently, the nuclear translocation of p65 nuclear translocation functions as a signal for NF-κB pathway activation via the previously mentioned method [44]. TLR2-mediated *S. aureus* inflammation has been shown to be influenced by the MAPK signaling pathway [45]. Our findings indicate that Lut suppresses protein phosphorylation in the NF-κB and MAPK signaling pathways, thereby reducing the inflammatory response in MAC-T cells induced by *S. aureus.*

## 5. Conclusions

According to this investigation, Lut prevented *S. aureus* from inducing an inflammatory response in MAC-T cells. This effect was partially attributed to the TLR2 signaling pathway’s attenuation. In particular, Lut inhibits the phosphorylation of p38, ERK, and JNK in the MAPK pathway and lowers the phosphorylation levels of IκBα and p65 in the NF-κB pathway, which in turn limited the synthesis of pro-inflammatory cytokines. These results collectively imply that Lut shows significant promise as an adjuvant treatment for *S. aureus*-induced mammary gland mastitis. Animal trials conducted in vivo can be used in future research to confirm this possibility.

## Figures and Tables

**Table 1:** Primers used for RT-qPCR.

Species	Gene Name	Primer Sequence (5′-3′)	GenBank Accession Number
Bos taurus	IL-1β	Sense: GGCAACCGTACCTGAACCCA	NM_174093.1
		Antisense: CCACGATGACCGACACCACC	
	IL-6	Sense: ATGCTTCCAATCTGGGTTCA	NM_173923.2
		Antisense: GAGGATAATCTTTGCGTTCTTT	
	TNF-α	Sense: ACGGGCTTTACCTCATCTACTCA	NM_173966.3
		Antisense: GGCTCTTGATGGCAGACAGG	
	MyD88	Sense: AGCAGCATAACTCGGATAA	NM_001014382.2
		Antisense: CAGACACGCACAACTTCA	
	IRAK4	Sense: TGGCAAAGACAGGACATCTG	NM_001075998.1
		Antisense: CACAACTCCCAAACCCTCCTT	
	IRAK1	Sense: GAGTTCCAACGTCCTTCTGG	NM_001040555.1
		Antisense: CTCCCGGTCTTCACGTACTG	
	TRAF6	Sense: CGGTGACTCTCTCCAGGTGT	NM_001034661.2
		Antisense: TGGACATTTGTGACCTGCAT	
	GAPDH	Sense: TGCTGGTGCTGAGTATGTGGTG	NM_001034034.2
		Antisense: CAGTCTTCTGGGTGGCAGTGAT	

## Data Availability

Data are contained within the article and Supplementary Materials.

## Supplementary Materials

The following supporting information can be downloaded at: https://www.mdpi.com/article/10.3390/vetsci12020096/s1, Figure S1: the original image of Figure 5a in the article. Figure S2: the original image of Figure 6b in the article. Figure S3: the original image of Figure 7 in the article.
