# Supplementary material for: Luteolin Alleviates Inflammation Induced by Staphylococcus aureus in Bovine Mammary Epithelial Cells by Attenuating NF-κB and MAPK Activation
Source: Vet Sci. 2025 Jan 27;12(2):96. doi: 10.3390/vetsci12020096 (PMC11861667; doi:10.3390/vetsci12020096)
Supplement: Supplementary file 1 [file vetsci-12-00096-s001.zip › vetsci-3352212-supplementary.pdf]

The following is Supplementary Materials:

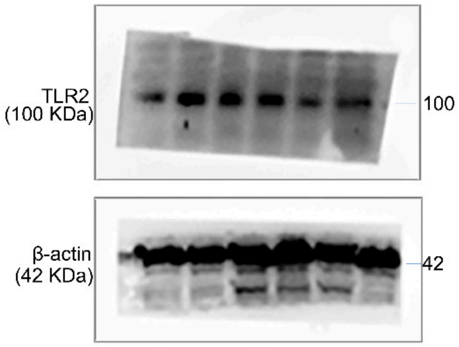

Figure S1 the original image of Figure 5a in the article

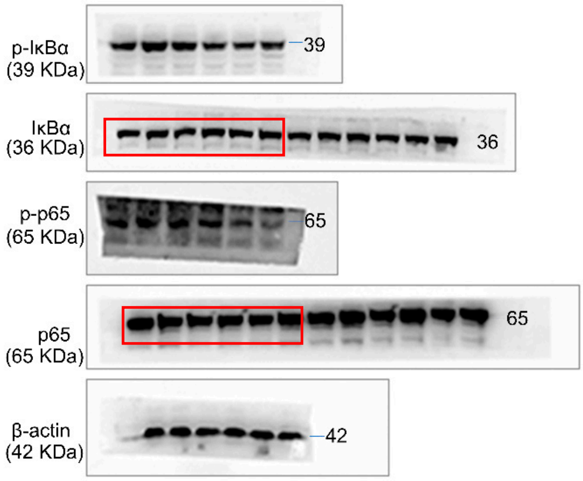

FigureS2 the original image of Figure 6b in the article

The red frame denotes the images utilized in this article.

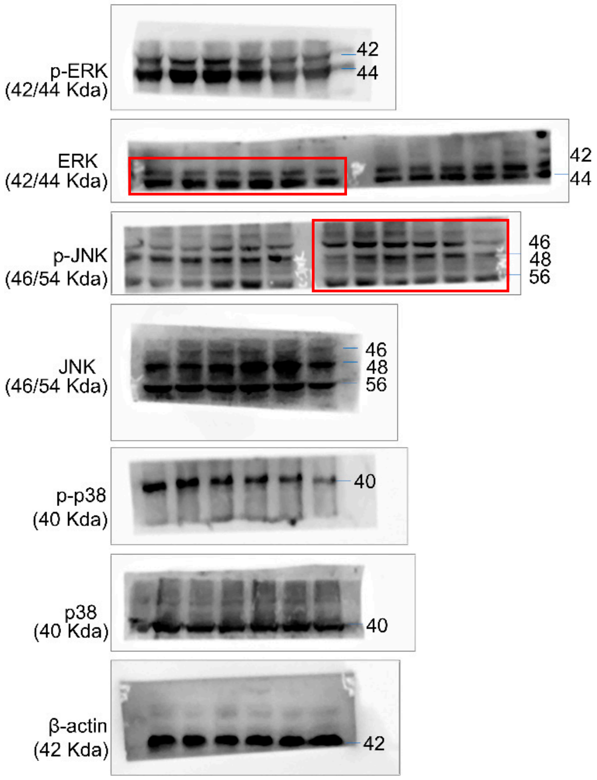

FigureS3 the original image of Figure 7 in the article

The red frame denotes the images utilized in this article.
